# Artificial intelligence methods applied to longitudinal data from electronic health records for prediction of cancer: a scoping review

**DOI:** 10.1186/s12874-025-02473-w

**Published:** 2025-01-28

**Authors:** Victoria Moglia, Owen Johnson, Gordon Cook, Marc de Kamps, Lesley Smith

**Affiliations:** 1 School of Computing, University of Leeds, Woodhouse Lane, Leeds, LS2 9JT UK; 2 Leeds Institute of Clinical Trials Research, University of Leeds, Clarendon Way, Leeds, LS2 9NL UK; 3 NIHR Leeds Biomedical Research Centre, Chapeltown Road, Leeds, LS7 4SA United Kingdom

**Keywords:** Machine learning, Health data, Longitudinal data, Cancer, Time-series, Temporal, Artificial intelligence

## Abstract

**Background:**

Early detection and diagnosis of cancer are vital to improving outcomes for patients. Artificial intelligence (AI) models have shown promise in the early detection and diagnosis of cancer, but there is limited evidence on methods that fully exploit the longitudinal data stored within electronic health records (EHRs). This review aims to summarise methods currently utilised for prediction of cancer from longitudinal data and provides recommendations on how such models should be developed.

**Methods:**

The review was conducted following PRISMA-ScR guidance. Six databases (MEDLINE, EMBASE, Web of Science, IEEE Xplore, PubMed and SCOPUS) were searched for relevant records published before 2/2/2024. Search terms related to the concepts “artificial intelligence”, “prediction”, “health records”, “longitudinal”, and “cancer”. Data were extracted relating to several areas of the articles: (1) publication details, (2) study characteristics, (3) input data, (4) model characteristics, (4) reproducibility, and (5) quality assessment using the PROBAST tool. Models were evaluated against a framework for terminology relating to reporting of cancer detection and risk prediction models.

**Results:**

Of 653 records screened, 33 were included in the review; 10 predicted risk of cancer, 18 performed either cancer detection or early detection, 4 predicted recurrence, and 1 predicted metastasis. The most common cancers predicted in the studies were colorectal (*n* = 9) and pancreatic cancer (*n* = 9). 16 studies used feature engineering to represent temporal data, with the most common features representing trends. 18 used deep learning models which take a direct sequential input, most commonly recurrent neural networks, but also including convolutional neural networks and transformers. Prediction windows and lead times varied greatly between studies, even for models predicting the same cancer. High risk of bias was found in 90% of the studies. This risk was often introduced due to inappropriate study design (*n* = 26) and sample size (*n* = 26).

**Conclusion:**

This review highlights the breadth of approaches to cancer prediction from longitudinal data. We identify areas where reporting of methods could be improved, particularly regarding where in a patients’ trajectory the model is applied. The review shows opportunities for further work, including comparison of these approaches and their applications in other cancers.

**Supplementary Information:**

The online version contains supplementary material available at 10.1186/s12874-025-02473-w.

## Background

Cancer is a leading cause of death globally and the burden of cancer continues to grow annually, with approximately 20 million cases and 10 million deaths reported in 2022, and cases expected to reach 35 million in 2050 [1]. Early diagnosis of cancers can lead to improved outcomes due to earlier intervention and a potentially increased range of treatment options [2, 3]. Prediction models aim to facilitate earlier diagnosis by finding patients at high risk of developing cancer, performing early detection of cancer, or by signalling patients at risk of metastasis or recurrence [4]. These models have the potential to influence future research, or to directly influence care through use as decision support tools. Traditionally, risk prediction models have been built using statistical methods, however, artificial intelligence (AI) has shown promise in improving the predictive capabilities of models [5]. AI is a set of technologies which aim to mimic human decision making [6]. More specifically, AI technologies are systems which are developed to provide an informative output based on human objectives [7]. Machine learning (ML) is a subset of this field which utilises algorithms that ‘learn’ from experience to optimize a defined task. While these terms have different meanings, within this field they are often used interchangeably given the subset of AI that is most appropriate for this application.

Cancer prediction models often utilise electronic health records (EHRs), which contain retrospective data collected by healthcare professionals during the course of a patient’s care, for example, laboratory tests or diagnoses. These are used to inform predictions as the records contain data that is routinely collected for patients. The increasing use and availability of EHRs provides a vast amount of data for prediction models and allows studies to be conducted retrospectively at a fraction of the cost of traditional prospective studies [8].

Many cancer prediction models use a cross-sectional approach to using EHR data, without considering the temporal aspect of the data; however, longitudinal data could be explored to fully exploit the information stored in EHRs [8]. Patients’ measurements should be viewed in context - changes over time may provide more information about a patients’ health than viewing static observations, and more recent observations may be more informative than more distant ones. For example, while long-standing diabetes is a risk factor of pancreatic cancer, new-onset diabetes has been suggested to be an indicator of asymptomatic cancer [9–11]. Quantities such as laboratory tests are also subject to inter-patient variability, and changes in these values may be more informative than instantaneous measurements [12]. Longitudinal data has been successfully used in other healthcare domains, such as mortality and sepsis prediction [13–15].

Problems requiring analysis of longitudinal data occur in many applications, such as meteorology, finance, transportation, and audio processing [16, 17]. These scenarios require specialized methods due to particular challenges introduced by the temporality, including correlation between consecutive inputs and high dimensionality where time points are treated as individual inputs. Time-series data in healthcare present specific additional challenges, including: data irregularity, as time-intervals between observations are often irregular; data sparsity, which occurs as a result of both infrequent healthcare interactions and one-hot encoding of categorical medical encounters; data heterogeneity, referring to highly diverse trajectories and outcomes of patients; and model opacity, as many models for time-series modelling require complex methods that are not interpretable [18].

A number of reviews have evaluated methods used for longitudinal health data [18–20]. Cascarano et al. provided a narrative review of ML methods that may be applied to longitudinal biomedical data [20] , while Carrasco-Ribelles et al. systematically reviewed studies that use longitudinal data from EHRs for AI-based prediction models [19] and Xie et al. systematically reviewed deep learning approaches for representing temporal data in health records. None focused on cancer and incorporating all possible methods, including deep learning and feature engineering approaches. In the current review, maintaining a focus on cancer prediction means the reviewed studies are addressing common data challenges; due to the long-term and progressive nature of cancer, they will generally rely on sparse data collected over a longer period of time.

In addition, the most recent systematic review identified had records published up to January 2022 [19]. In such a fast moving field, it is likely that the methods used have developed in the two years before the current work. Cascarano included work published up to 2023, however this review was not performed systematically [20].

To fully realise the potential of cancer prediction models in improving cancer outcomes worldwide, prediction models require rigorous methods in both development and validation. To date, no reviews have examined the quality of studies investigating the use of longitudinal data for cancer prediction.

This review aims to provide a summary of AI approaches that have been used to predict cancer from longitudinal data in EHRs. To this end, a scoping review was conducted [21]. The objectives of the review are as follows:

1. Identify and summarise approaches used for prediction of cancer from longitudinal data stored in EHRs.
2. Evaluate time windows used within prediction models against a framework.
3. Identify common areas where risk of bias is increased in cancer prediction research to guide future research using the Prediction model Risk Of Bias ASsessment Tool (PROBAST) [22].
4. Provide recommendations for design and reporting of longitudinal prediction models for cancer.

## Methods

This scoping review followed the Preferred Reporting Items for Systematic Reviews and Meta-Analyses extension for scoping reviews (PRISMA-ScR) [23].

### Databases and search strategy

The search strategy was developed iteratively by the authors. Six databases were searched: MEDLINE, EMBASE, Web of Science, IEEE Xplore, SCOPUS, and PubMed. The search strategy was adapted to be appropriate for each database, however, each search included terms relating to each of the concepts relevant to the scoping review question. These concepts were “artificial intelligence”, “prediction”, “EHRs”, “longitudinal”, and “cancer”. Full search terms are provided in Additional File 1. The search was conducted on 15^th^ August 2023 and updated on both the 2^nd^ February 2024 and the 9^th^ August 2024 with no limitation by year.

Citations and reference lists were searched for each of the eligible studies to retrieve additional records that were not retrieved in the initial search.

### Eligibility criteria and study selection

All records were imported to Rayyan, a platform developed to support systematic reviews, and duplicates were removed. The titles and abstracts were then screened by V.M and evaluated against the exclusion criteria. Where there was ambiguity within the inclusion criteria, records were discussed with L.S. and O.J. until a decision was reached. The resulting records were screened for full-text eligibility.

Eligible studies were those using longitudinal data from EHRs to predict cancer, including prediction of metastasis and recurrence. The EHR data was not restricted to structured data.

Articles were excluded based on the following criteria:

1. Not a primary research article.
2. The study does not predict cancer.
3. The study does not use AI/ML methods.
4. The study does not use longitudinal predictors.
5. The study is purely an implementation or validation study.
6. The cancer predictions are not patient level.
7. The method does not predict a specific outcome (i.e., clustering or phenotyping studies are not eligible).

Longitudinal predictors were defined as those allowing some representation of change between different time points, i.e., simply using the maximum value of a blood test within a time window was not considered to be longitudinal. Not all predictors were required to be time-varying. Methods were considered to be AI or ML methods if the authors described the methods as such, or if the methods were more complex than standard statistical methods such as logistic regression.

### Data extraction and synthesis

Data were extracted relating to several areas of the articles: (1) publication details, e.g. author, journal, date; (2) study characteristics, including outcome of interest, study design, population and setting, and sample size; (3) input data, including data type and fields used; (4) ML methods, including any feature engineering, the models used, evaluation metrics, and validation methods; (5) reproducibility, including data and code availability; and (6) quality assessment using the PROBAST framework [22]. The PROBAST tool is a framework for evaluation of Risk of Bias in prediction models. This tool is primarily aimed at statistical models; an AI extension is being developed, however, there is not currently an equivalent tool for AI models [24]. Therefore, the original PROBAST tool was used. Full extraction tables are provided in Additional File 2.

### Terminology

**Fig. 1:**
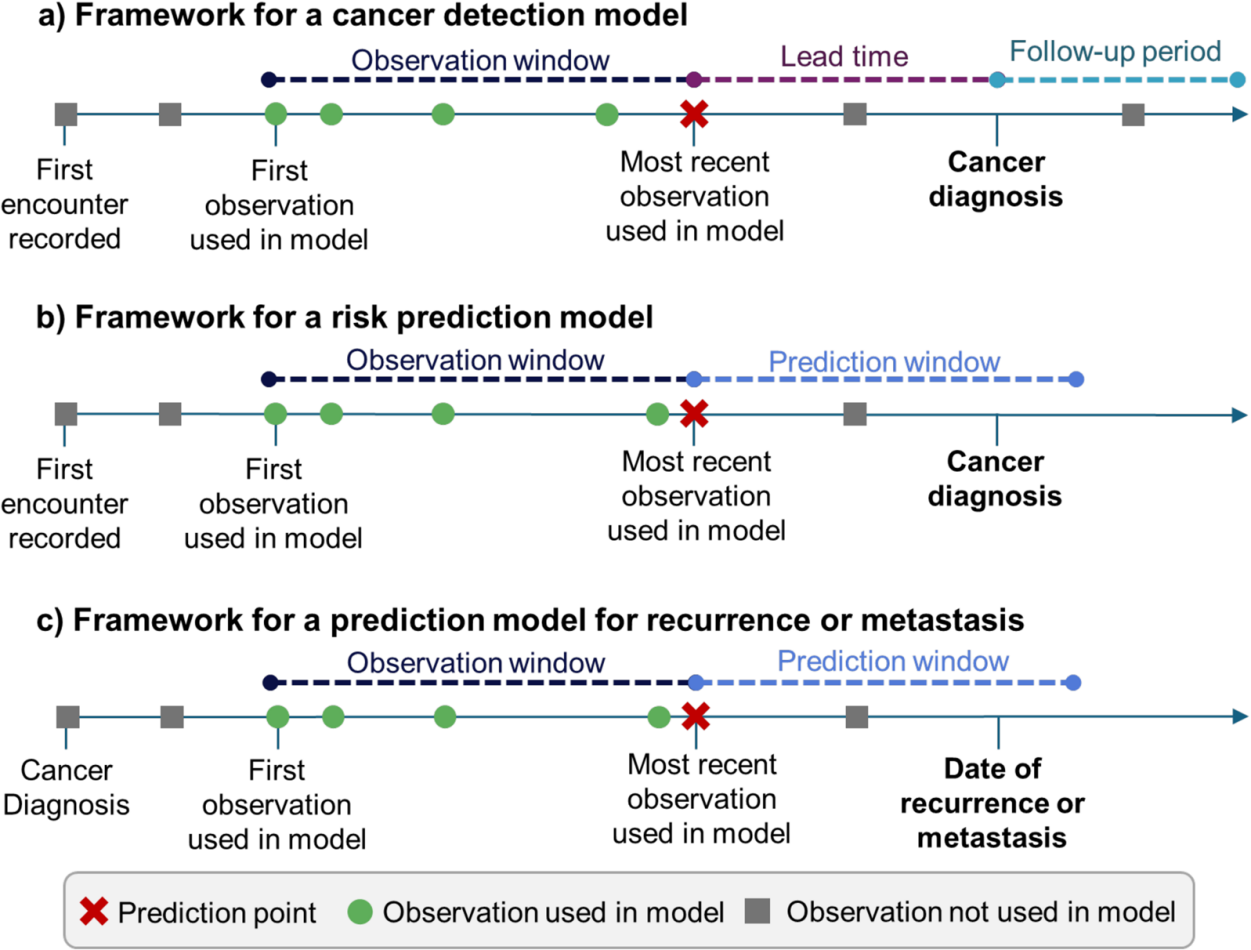
Definition of terminology used in this review to describe different time windows relevant to prediction models using longitudinal data

To assess the models used by studies in the review, we have used a modified version of the taxonomy used in [25] to define specific intervals relevant to each type of model: cancer detection models, risk prediction models, and metastasis/recurrence prediction models. This taxonomy is demonstrated in Fig. 1.

Cancer detection models (Fig. 1a) use the diagnosis date of a cancer patient and define an index date for controls. Data before the diagnosis or index date, within the observation window, is used to predict the outcome at that point. Early cancer detection models should also have a lead time, which is a gap between the last measurement in the model and the outcome. Data within the lead time window are not used in the model. Cancer detection models can include follow-up period to reduce bias: this reduces the possibility that a control has an undetected cancer.

Risk prediction models (Fig. 1b) aim to find high risk populations. They often define a study-wide time-frame, the prediction window, and use data before this window to predict the risk of a patient developing cancer within this window.

Models that predict recurrence or metastasis are more varied; they may use surveillance data from the initial diagnosis of cancer to predict the likelihood of recurrence or metastasis in the future (Fig. 1c). They may also use data from before the diagnosis.

A more detailed framing is provided in [25], however for this review less granular classifications were required.

## Results

### Retrieved studies

**Fig. 2:**
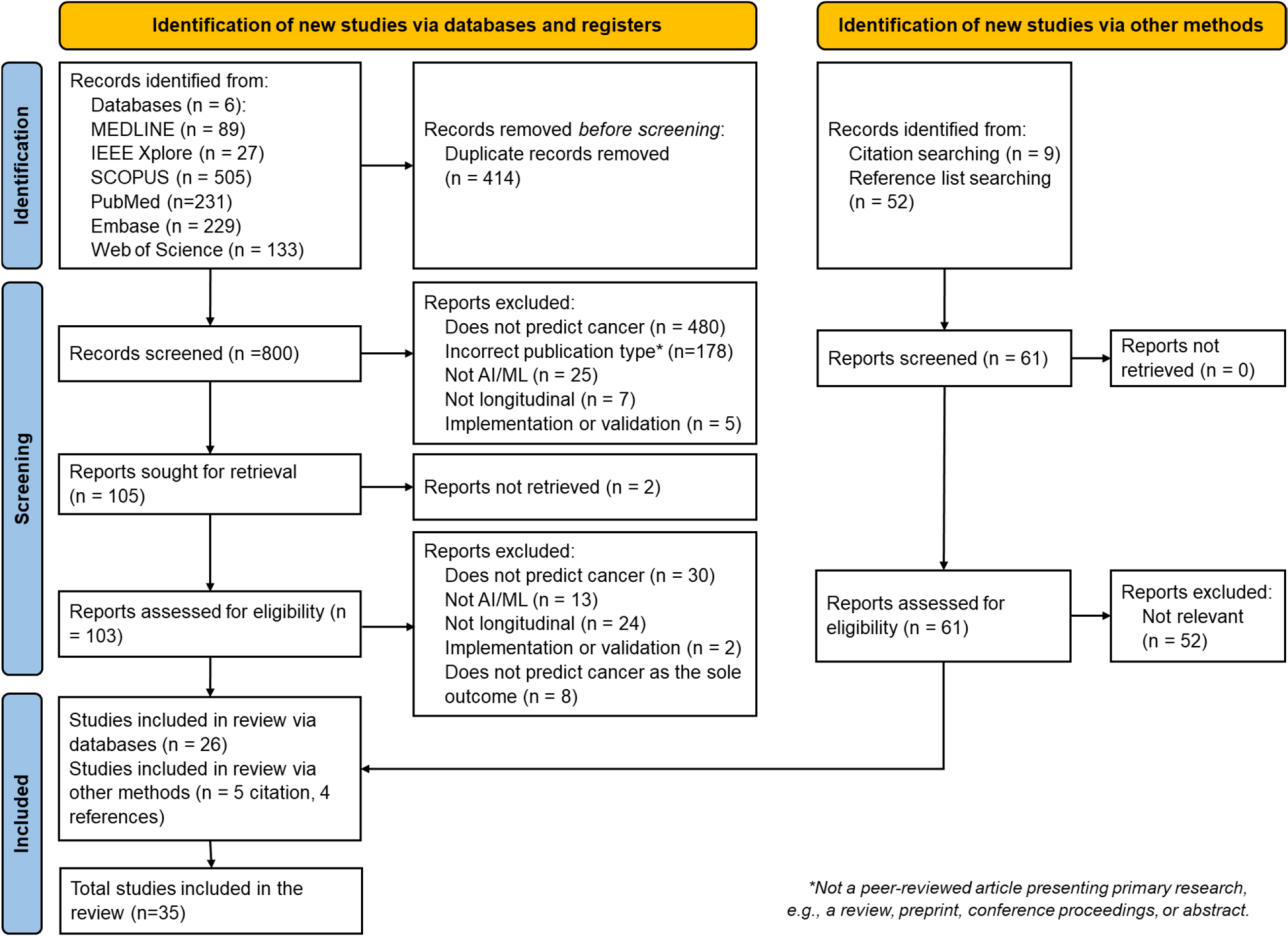
Flow diagram for study identification and selection. Developed using the PRISMA template provided in [21]

**Fig. 3:**
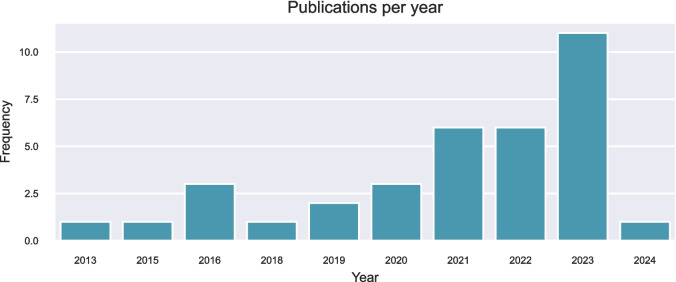
Number of retrieved studies by year of publication. *Year to date 09/08/2024

Searching the 6 databases returned 1214 studies, of which 414 were duplicates. A further 61 studies were retrieved from reference and citation searches. Following screening and eligibility assessment, 35 studies were included in the final review. A flowchart showing the selection process is provided in Fig. 2. The number of studies published by year is shown in Fig. 3.

### Study characteristics

#### Study setting and population

Studies used populations from the USA (19, 54%), the Netherlands (4, 11%), Taiwan (5, 14%), Denmark (2, 6%), Sweden (1, 3%), South Korea (1, 3%), Israel (1, 3%), and Singapore (1, 3%). One study did not report where the population originated from, and one study used an additional dataset from the UK as a validation set. Five studies (14%) used single-centre data for model development, 20 (57%) used data from multiple centres linked by location or healthcare provider, nine studies (25%) used nationwide datasets, and one study (3%) did not report the study setting. The nationwide studies originated from Sweden, Taiwan, South Korea, and Denmark, while the multi-centre studies using data from affiliated practices originated in the USA (*n* = 15), the Netherlands (*n* = 4), and Israel (*n* = 1). The studies used case–control (9, 26%), nested case–control (6, 17%), or cohort (20, 57%) study design. Of the settings for the datasets used, four studies used primary care (11%), seven used secondary care (20%), 23 used both primary and secondary care data (66%), and one did not report.

#### Outcomes/Prediction Task

Ten studies predicted the risk of cancer within a specific time-frame. Twenty studies focused on either detection or early detection of cancer. One study predicted metastasis and four studies predicted recurrence.

The most common cancers included in the studies were pancreatic and colorectal cancer (both 9 studies, 26%). There were 6 studies predicting lung cancer (17%), 3 studies (9%) each considering liver and gastric cancer and 2 (6%) considering breast, skin, leukaemia, and oesophageal cancer respectively. Brain metastasis, cancer of the small intestine, anal cancer, cervical cancer, and prostate cancer were each predicted in one study respectively. Additionally, one study predicted cancer as a generic outcome, with no site specified. Note that some studies developed models for multiple sites.

#### Clinical features

**Fig. 4:**
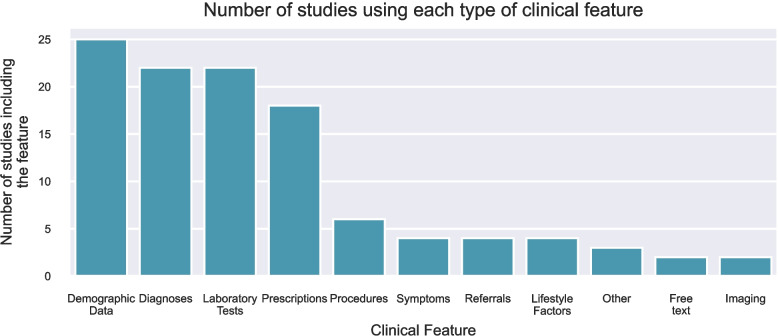
Frequency of studies using each type of clinical feature, where the total number of studies is 35. The category ‘Other’ includes studies using images, histology features, and tumour staging

The most commonly included features were demographics (25, 71%), diagnoses (22, 63%), laboratory tests (22, 63%), and prescriptions (18, 51%). Other features included symptoms, referrals, procedures, free text notes, lifestyle factors, images, tumour staging, and histological features. Frequency of the features is shown in Fig. 4.

The clinical variables selected varied between the approaches taken. All of the studies using feature engineering used laboratory tests in their models, whereas only a third of the models using sequential inputs did the same. In addition, all but one of the feature engineering models used demographic data, in contrast to around two-thirds (*n*=12) ofthe sequential input models.

### Model characteristics

Methods for representing temporal information within predictive models can be divided into two main approaches. The first approach utilises feature engineering, a process where data are extracted and manipulated to form informative variables, to find meaningful representations of temporal data or capture key temporal characteristics. These features, generated for each patient, can be used as inputs in downstream AI models to generate an individual classification or risk score. For this approach, we analyse the methods for representing the longitudinal information, rather than the subsequent AI model as these are generally not directly tailored to handle longitudinal data. The second approach uses temporal sequences as direct inputs. This approach generally includes a model that has specific mechanisms to model the sequential nature, namely the dependence between time steps.

#### Feature engineering for representation of sequential data

Sixteen models used feature engineering for the representation of temporal data. Various approaches were taken; these are summarised in Table 1.

**Table 1:** Feature engineering methods used by studies in the review

Name of Method	How it works	Advantages	Limitations
Trend Features	Represent change between numerical features by calculating the slope between time points	Simple method for representing change. If paired with an interpretable ML method, can be easily understood	Treats change as linear, may not capture more complex behaviours. Cannot be used for categorical features
Absolute change	Use change between two defined time points as input. Sometimes calculated using estimated values at the time points, for example by using linear regression with existing measurements	Simple method for representing change. If paired with an interpretable ML, can be easily understood	Only useful for numeric features, cannot be used to represent sequential diagnoses for example
Summary statistics of variation	Summarise most significant changes, e.g. total variation, largest increase	Less likely to be affected by noise due to intra-subject variability	Does not give an indication of how quickly changes occurred. Cannot be used for categorical variables
Laboratory test dynamics	Represent dynamics by calculating doubling time and velocity from two consecutive data points	Used in clinical research for PSA analysis [4].	Only useful for numeric features, cannot be used to represent sequential diagnoses for example
Pattern Mining	Find common predictive patterns by defining possible patterns iteratively—e.g. diagnosis 1 before diagnosis 2. Use binary indicators of presence of patterns as a feature	Can be used for numerical and categorical variables. Easily interpretable features(depending on the model used)	Use for numerical values (e.g. laboratory values) requires categorising them into low, medium, and high which may introduce bias
Signal decomposition	Decompose the time series into meaningful features using signal processing transforms such as the wavelet and Fourier transforms	Commonly used features in signal processing	May require denser data than is present in many EHRs
Unsupervised methods	Deep learning methods to find generalised representations of the data. Use methods such as autoencoders which learn by iteratively reducing and reconstructing a signal, minimizing the reconstruction error. The learned reduced representation can be used as an input to other classification models	The features learned are not optimised for a single task and can therefore be used for other downstream tasks. Does not require human knowledge to design and select the features, and therefore can reveal previously unknown features	Difficult to train. General representations may include irrelevant features for the task, adding increased complexity without added benefit

Three studies used ‘trend’ or ‘slope’ features [26–28]. Five used the absolute change between defined time points, for example, the 12 month change before prediction date [29–33]. For two of these studies [29, 30], the values used to calculate the absolute change were inferred from models trained on each patients individual trajectory for that variable. Kinar et al. [29] fit linear regression models which were used to predict values at 18 and 36 months before to the index, and the change between those time points was used as the trend feature. A similar approach was used by Rodriguez et al. [30], fitting a linear mixed effects model to log-transformed laboratory measurements to provide a ‘smoothed’ trajectory (i.e., with reduced measurement error). Predictions were then used to calculate the 6-month change in log-transformed measurement.

Rubenstein et al. used the largest increase and total variation of a measurement as features [34]. Labaratory dynamics were used by Beinecke et. al. [35] One study stated ‘trending’ features were used, but did not describe how these were calculated [36]. In three studies, pattern mining was used to find predictive temporal patterns [37–39]. One study applied Wavelet and Fourier transforms to longitudinal data and used the coefficients as features to a model [40].

Unsupervised approaches were also used to extract features from time-series. Lasko et al. and Ho et al. both use autoencoders to learn general representations of a patient’s trajectory [40, 41].

#### Models taking sequential data as direct input

Twenty studies used deep learning models with a sequential input, either the raw sequence or ‘binned’ into discrete time intervals. The methods used are summarised in Table 2.

**Table 2:** Summary of methods used by studies in the review which take a sequential input

Name of method	General Idea	How it works	Advantages	Limitations
Neural networks	Inspired by `neurons' in the brain, pathways learn from training data	Each time step is a `node' to the network Weights in hidden layers are learned iteratively by updating them to reduce prediction error [42].	Can model non-linear relationships well	Assumes independence between nodes; this is unrealistic for time series. The weights are different at each node, meaning a pattern occurring in different parts of a sequence may not be captured
RNN based models	Methods developed in text processing which have adaptations making them appropriate for sequential data	These methods model dependence between sequential inputs. Vanilla RNNs struggle with long-term dependencies, LSTMs and GRUs are adaptations with mechanisms to address this [43].	Shared parameters across each layer mean a concept can be captured regardless of its position within the sequence. Dependence between timesteps is more realistic than assuming independence	Require inputs of equal length, meaning patients' data may have to be padded to reach maximum length
CNNs	Originally developed for images, they use filters to find key features	Learned filters scan along the input and are activated by key features. Longitudinal data are represented as 2D images with dimensions time x feature, or different 1D filters are learned for each feature individually [44].	Features are captured regardless of their place in the sequence	Require inputs of equal length, meaning data must be padded (e.g., with zeros). The patterns found are localised—longer scale patterns may be missed
CNN-LSTM	Process spatial information before feeding features into an LSTM to process the temporal dimension	The CNN layers reduce the dimensions of the time series input, usually for spatial data (e.g., images). The LSTM network then models longer-term dependencies of the temporal dimension	Combines spatial and temporal models to model time-series of spatial inputs	Can be computationally expensive and slow to train
Transformer	Developed in text processing, they aim to model context in sequences	Use `attention'—a mechanism that places more emphasis on parts of the sequence that provide context for related sections of input. Temporality is not inherent, this is introduced via positional encoding [43, 45].	Transformers are designed for parallel computing, meaning they scale more efficiently than RNN based models	Requires a large amount of data to train. Complexity depends quadratically on the sequence length

Ten studies used models based on recurrent neural networks (RNNs): seven used long Short-Term memory models (LSTMs) [40, 46–51] and seven used gated recurrent units (GRUs) [40, 46, 49, 50, 52–55]. Two studies [53, 54] used the reverse time attention model (RETAIN) proposed by Choi et al. which introduces an attention mechanism to a GRU to prioritise the most meaningful visits in a patient’s input sequence [56].

Five studies used convolutional neural networks (CNNs) [40, 57–60], while one study [61] used a CNN-LSTM, representing diagnoses and medications as a 2D matrix and performing 2D convolutions over the input.

Three studies [62–64] used a standard feed-forward neural network, where each time-step was represented by a node in the architecture. In two of these, Park et al [63, 64] trained a separate neural network for each variable as an ‘embedding network’ to reduce the dimensionality of the input, and these reduced features were concatenated to form an input to a final classification network.

Six studies utilised transformer architectures [40, 49, 54, 55, 61, 65]. Positional encodings were derived in one study using the common approach of evaluating sinusoidal functions of varying frequencies at the point the token appears in the input sequence [40]. Two studies adjusted this approach so that the sinusoidal functions were evaluated at a patient’s age, rather than the position within the sequence [49, 55]. Rasmy et al. introduce multi-layered embeddings for position, denoting not only the order of visits, but also the order of codes within the visits [54]. Two studies did not report the method of position embedding [61, 65].

The deep learning methods used require inputs of uniform length. There were a number of approaches to addressing missing data along the temporal axis. Five studies had categorical features representing the presence of an event within a specific window, hence the length of inputs did not need specific attention [51, 58–61]. Where events are represented as embeddings or numerical values are used, any sequence that is shorter than the maximum sequence length must be coerced in some way. Five studies [40, 47, 48, 50, 65] ‘padded’ the input by adding zero vectors to the sequence. Three studies [46, 52, 57] used forward-filling, where missing data along the temporal axis is filled by using the most recent present measurement. Six studies did not report the method of addressing input sequence length [54, 55, 62–64].

#### Prediction windows

The prediction windows for each model, as defined in 2.4, are shown in Tables 3 and 4. Table 3 shows the time windows used in each of the risk prediction models. For the observation window, all but one of the risk prediction studies used the full available data within the study period and did not impose any limit on data before the index date. The prediction windows varied between 3 and 60 months, with 36 months being the most common. Only one of the risk prediction studies investigated multiple prediction windows [55]. This study presented the majority of their results with respect to the 36-month prediction window, stating that it is a reasonable window for screening.

**Table 3:** Time windows used in risk prediction models

Study	Cancer Type	Observation window (months)	Prediction window (months)
[57]	Skin	Full study period	60
[62]	Any site	36	48
[52]	Liver	Full study period	36
[49]	Liver	Full study period	36
[32]	Pancreatic	Full study period	18
[33]	Pancreatic	Full study period	36
[31]	Pancreatic	Full study period	18
[55]	Pancreatic	Full study period	3, 6, 12, 36, 60
[28]	Pancreatic	Full study period	18^a^
[61]	Lung	Full study period	36

^a^The risk prediction window starts 6 months after the prediction point

**Table 4:** Time windows used in each of the cancer detection models

Study	Cancer Site	Observation window (months)	Lead time (months)	Follow up time (months)
[46]	Colorectal	Full history	0, 12, 24, 36	0
[29]	Colorectal	Full history	3–6	Unspecified
[37]	Colorectal	6	0	Unspecified
[38]	Colorectal	6	0	Unspecified
[58]	Colorectal	36	12	Unspecified
[39]	Colorectal	6	0	Unspecified
[47]	Gastric	Unreported	0	Unspecified
[26]	Luminal GI	36	6, 12, 36, 60	0
[34]	Oesophageal, Gastric	48	12	Unspecified
[41]	Leukaemia	Full history	0	0
[51]	Leukaemia	12	6	Unspecified
[63]	Pancreatic	Full history	0, 3, 6, 12, 24, 36	Unspecified
[64]	Pancreatic	Full history	0, 3, 6, 12, 24, 36	Unspecified
[54]	Pancreatic	Unreported	0	Unspecified
[27]	Pancreatic	36	0	36
[50]	Breast, Lung, Cervix, Liver	N/a^a^	0	0
[36]	Lung	60	3–6, 9–12	Unspecified
[65]	Lung	60	0	36
[59]	Lung	36	12	Unspecified
[60]	Skin	36	12	Unspecified

^a^Defined by number of visits, not time-period

Table 4 shows the time windows for the 20 cancer detection models. Five of these used the full history of the patient as the observation window. The observation windows of those studies that restricted the window ranged between 6 and 60 months. Two studies did not report their observation window. One study did not define their observation window by time period, but rather by number of measurements. Nine of the studies have no lead time, instead detecting cancer using all data that was available before cancer diagnosis. Eleven studies investigated early detection of cancer using a lead time, these varied between 3 and 36 months. Five studies investigated numerous lead times. Follow-up time was poorly reported in most studies, with 14 studies not providing information on this window. Four studies did not follow-up controls for diagnosis, while two studies gave a follow-up time of 36 months.

The windows for metastasis and recurrence prediction models are shown in Table 5. One study included follow up of controls [53]. Two studies defined the start of the observation window as a specific clinical event relating to the primary cancer [35, 40].

**Table 5:** Time windows used in metastasis or recurrence prediction models

	Observation Window	Prediction Window	Follow-up
[35]	From date of initial diagnosis to date of recurrence diagnosis	Unclear	-
[53]	Unclear	Unclear	12 months
[30]	6 months	12 months	-
[48]	Defined by number of observations	12 months	-
[40]	From date of surgery to date of recurrence diagnosis	Unclear	-

#### Comparison to cross-sectional models

Of the studies included in this review, seven compared longitudinal methods to cross-sectional approaches, using data from only a single timepoint [26, 37–39, 52]. Ioannou et al. reported improvement in discrimination and calibration over cross-sectional results when using the sequential input model, but no significant improvement using engineered features [52]. Kop et al. found in one study that engineered temporal features improved predictions [37], but this result was not repeated in later work [38]. Hoogendoorn et al. did not report an improvement in predictive performance, but did observe that performance was more stable across different data types in sensitivity analysis [39]. Read et al. noted a trend towards improvement, but the results were not definitive [26]. In the multimodal study by Li et al., longitudinal was found to improve predictions in modalities integrating both image data and clinical data, but not in all modalities [65].

#### Explainability

Twenty-two studies considered either explainability of predictions or model reasoning. Thirteen of these (10 feature engineering models, 3 sequential input models) presented model level interpretability such as feature importances to demonstrate what information is used by the model to make predictions. Nine studies (1 feature engineering model, 8 sequential input models) had prediction level explanations, where the factors contributing to an individual prediction are calculated. The methods used for individual prediction explanations were local interpretable model-agnostic explanations (LIME) [47], attention-based interpretation [49, 53, 54], integrated gradients [55, 61], and Shapley additive explanations (SHAP) [34, 36, 53, 64].

### Reproducibility of research

Thirteen studies (36%) had code available to use online. Two studies used data that was adapted to a common data model: Kim et al. used the Observational Medical Outcomes Partnership Common Data Model (OMOP-CDM) [47], and Jia et al. [28] used data adhering to the TriNetX standard data model [66]. One study [47] used data which is freely available online. Fifteen studies used datasets which can be requested or purchased: the Veterans’ Affairs Corporate Data Warehouse [31, 32, 34, 52, 55], the Kaiser Permanente Southern California databank [30–33, 36], the Julius General Practitioner Network [38, 39, 46], Cerner Health Facts [53, 54], HCUP State Inpatient Databases (SID) [50], IQVIA datasets [51], and TriNetX [28]. Six studies used data that is available to researchers within the country of origin only [27, 55, 58, 59, 61, 62].

### Quality Assessment

**Fig. 5:**
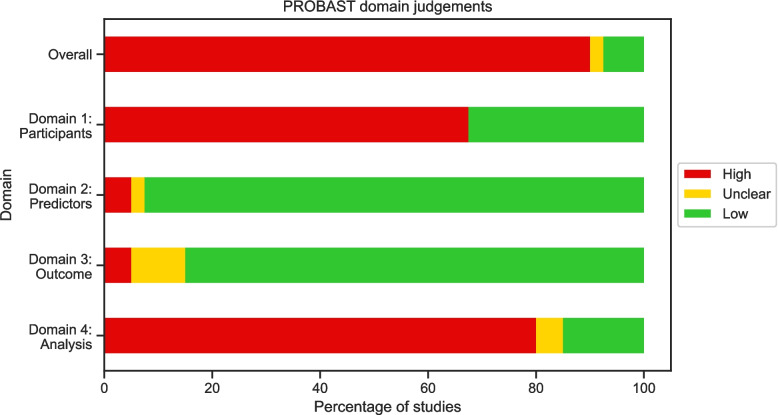
A summary of risk of bias judgements assessed using the PROBAST framework. Note that some studies may have multiple risk of bias assessments where external validation was performed or the study included more than one predictive model

An overview of the domain judgements for the PROBAST assessment are shown in Fig. 5 and individual judgements are provided in Additional File 2. The overall risk of bias was high for 90% of the studies in the review, low for 7.5%, and unclear for 2.5%.

## Discussion

This scoping review analysed AI methods applied to longitudinal EHRs for the prediction of cancer. A range of approaches were identified for prediction of cancer from longitudinal EHRs. These approaches were categorised into those using feature engineering for representation of temporal data and those using a sequential input directly.

The review highlights common methods and feature sets used the field, and also the lack of consistency in prediction windows between studies.

### Main findings of the review

Nationwide studies were conducted in Sweden, Taiwan, South Korea, and Denmark, however these studies are not currently possible in many countries due to the lack of national datasets. All studies using data from multi-centre organisations originated in three countries: the USA, the Netherlands, and Israel. There are benefits to using multi-centre data and nationwide data, including larger datasets and increased generalisability of results, however researchers are often limited by the availability of appropriate datasets [8].

The challenges of using EHR data for prediction models are well documented [8], primarily relating to data quality issues and inconsistent recording between different clinicians and sites [67]. Recording of data may also change over time within a healthcare centres, therefore additional care should be taken when developing longitudinal models to ensure models are robust to temporal shift [68]. While EHR poses extra challenges to analysis, if models are intended for use within EHR systems they are likely to encounter the same quality issues. Consideration of these aspects in model development should make the resulting algorithms more robust to similar issues upon deployment. EHR data provides numerous benefits over prospectively collected data as it is more reflective of clinical practice and is not as expensive or time-consuming to collect.

The intended use case of models is a key consideration when selecting data sources. If a model is intended to be used for early detection, this should be reflected in the dataset by utilising data that would be available at the point of use. Where studies are to use linked primary and secondary data, it should be considered whether these data would be linked in practice as this has implications for clinical applicability. However, proof of concept research demonstrating improved disease detection from linked data can still be valuable as it provides motivation for cohesive electronic health record systems across healthcare networks and many countries are aiming towards linked health data in practice.

The most frequently considered cancers were colorectal and pancreatic, accounting for more than 50% of the included studies. These are likely commonly chosen due to the impact they have globally; colorectal cancer is the third most common cancer and the second leading cause of cancer death. Pancreatic is less common, ranking around 12th, but contributes to the 6th largest number of deaths, and is known to be difficult to diagnose. There is an unmet need for earlier diagnosis of rarer cancers. Although more data is available for patients with more common cancers, there is an opportunity to establish methods on those datasets so they can then be implemented and optimised for rarer cancers.

The choice of features has an impact on the choice of model and vice versa—many of the approaches to feature engineering shown in Table 1, such as trend features and signal decomposition, would not be appropriate for categorical information such as diagnoses. Similarly, approaches to missing data differ between different types of variables; for categorical features, where the feature indicates whether the feature was present or not at that time, missing data do not need addressing, whereas for numerical features such as laboratory tests missing data must be imputed. This is particularly a problem in models requiring fixed inputs length inputs such as RNN based models and CNNs.

The methods identified in this review are summarised along with their advantages and limitations in Tables 1 and 2 respectively. The most commonly used feature engineering method was absolute change in measurement, which is likely commonly chosen due to the ease of computation but requires expert knowledge to determine which times to calculate change between. The most common approach using sequential inputs was to use models based on RNNs. A general advantage of feature engineering is that the features can be used in relatively simple artificial intelligence algorithms, reducing the computational cost, although they require human input in crafting meaningful features. Alternatively, deep learning approaches have the capability to learn hidden patterns without the need for explicit crafting by an expert, including potentially undiscovered predictors. This gives rise to a key question; does added complexity increase accuracy, and does this increase justify the increase cost. The two approaches are rarely compared, and future research should aim to do this.

In addition, research should consider whether longitudinal data does improve the predictive capability of models. Few studies in this review compared longitudinal models to cross-sectional approaches, and those that did were not definitive in finding an improvement in performance although there was weak evidence to support an improvement, and no studies reported that longitudinal data harms predictions. Given the additional complexity and cost of incorporating longitudinal data, the question of whether this is justified should be considered.

As previously described, longitudinal data in healthcare provide specific challenges for prediction models. The methods found in this review address these in varying ways. Data irregularity was commonly addressed in feature engineering models by modelling patients’ trajectories individually to infer values at specific time-points or by calculating slopes from available data. Sequential methods often coded the relative times of observations to provide context to the models or required direct imputation of missing data in the temporal axis. Data opacity was considered in a number of studies aiming to develop explainable methods. The level of explainability achieved by models varied by the approach taken; feature engineering models were more likely to provide model level explanations, which are often simple to implement. However, these may not be as useful for clinicians as prediction level explanations, which can help a user understand why the model classified a patient a certain way, but do require more complex methods to implement, increasing the computational cost of a model.

Risk prediction models were evaluated against the longitudinal model framework described in Fig. 1. All risk prediction models except one used the full study period as the observation window, and no studies evaluated models using different observation windows. In risk predictions studies, this was generally a universal time window for the entire cohort, for example, from 2003–2011. Using all available data as the observation window may result in better performance as the full history is used, providing more context for patient data. Conversely, using all available data may hinder performance, by introducing additional noise into models. In addition, using longer time sequences may increase complexity of models and increase computational expense. Given this potential trade-off, studies should aim to evaluate the impact of various observation windows on model performance. Similarly, only one study experimented with different prediction windows. The risk prediction windows used by other studies varied significantly, even when predicting the same cancer, suggesting there is not clear window that should be assumed without investigation.

Cancer detection models should report three quantities: the observation window, lead time window, and follow-up time. A number of studies used the full patient history as the observation window, which has the potential to introduce bias to models as cancer patients may have systematically shorter trajectories as a result, which may be detected by sequential input models. Potential bias should also be mitigated by ensuring there is sufficient follow up of the control population, as patients may have been diagnosed with the cancer of interest at a later date, indicating a present but as yet undiagnosed cancer. Only one study reported including any follow up time [27]. Lead time is a key parameter to consider in early detection models. Most early detection studies experimented with different lead times, which allows for interpretation of how prediction accuracy changes with distance from the event of interest.

In general, the reporting of time-windows was poor in metastasis and recurrence prediction models. This makes it difficult to not only assess potential bias in the models, but also makes the intended use-case unclear, i.e., where would the prediction be made and how would this aid a clinician. As previously explained, follow-up time should be reported in studies predicting recurrence or metastasis to rule out potentially undiagnosed patients and hence mislabelled occurrences.

Given that current research into the use of longitudinal health records is in the early stages and studies are generally proof-of-concept, the reproducibility of the research is vital to ensure future work can build upon findings. Despite this, only around a third of studies included in the review have code that is available. Due to the confidential nature of health data, open access data is rare, however the availability of commercial datasets such as those used by studies in this review provides the opportunity for comparative works. For research using these sources it is especially important to be clear about how cohorts were selected. Clear reporting of methods and study setting is vital for reproducibility. The recent publication of an AI extension to the Transparent Reporting of a multivariable prediction model for Individual Prognosis or Diagnosis (TRIPOD-AI) statement provides a checklist of reporting items that should be followed by predictive models using AI [69].

The PROBAST assessment showed that most studies were at high risk of bias, with only three studies achieving low risk of bias overall. This is unsurprising given that the studies are generally exploratory, however the results highlight the common areas where risk of bias is introduced. The highest risk domains were domains 1 and 4, concerning study participants and analysis respectively. High risk in domain 1 was introduced due to case–control study designs and restrictions on participants (e.g., age based) without acknowledgement of how this affects the applicability of the model. These factors may be unavoidable due to data access restrictions; however, researchers should make the potential impact on the risk of bias clear and nested case–control studies should adjust for outcome frequency as described in the PROBAST framework [22]. In domain 4, common areas introducing risk of bias were an insufficient number of participants with the outcome, lack of follow-up periods for controls, inappropriate performance measures, and no accounting for overfitting. While low numbers of patients with the outcome is often determined by available data, the remaining areas can be mitigated by researchers through the following actions: ensuring follow-up of controls; reporting comprehensive performance measures, including both discrimination and calibration measures; and using cross-validation or bootstrapping to account for overfitting in the model.

### Recommendations for future work

In conducting this review, we identified several common areas for improvement which future work should aim to address:

1. Models should be reported using clear terminology, provided here or by Lauritsen et al. [25] Given the clinical application, it is especially important to clearly explain at which point the prediction model would be used, and which data would be available.
2. Given the lack of consensus on appropriate prediction windows for each of the cancers, studies should evaluate models at various time points to assess the optimal time windows for prediction. In addition, models should be evaluated against cross-sectional methods. It is not a given that adding longitudinal data improves performance, but it is likely to increase complexity. Researchers should aim to evaluate whether this added expense is justified for the problem.
3. To ensure reproducibility of research, studies using AI for prediction of cancer should adhere to the TRIPOD + AI statement to ensure methods are transparent [69]. Where possible, data should be made available for sharing. As this is often not appropriate for patient data, reporting of datasets should be comprehensive. Researchers should make the code used in the research publicly available.
4. When conducting cancer prediction research, researchers should be mindful of how bias may be introduced to the model. The forthcoming PROBAST-AI will provide guidance [24]. Mitigation strategies include ensuring sufficient follow-up of controls; reporting a variety of performance measures, including discrimination and calibration; and accounting for optimism and overfitting in the model using cross-validation or bootstrapping.

### Strengths of the current study

This review has multiple strengths. Firstly, the scope of the review covered all longitudinal methods to include a wide range of methodologies, found through an exhaustive search strategy. In addition, the review adheres to the PRISMA guidance for conducting scoping reviews. The review also provides a PROBAST assessment, highlighting common areas where risk of bias is high in longitudinal models.

### Limitations of the current study

This review has four main limitations:

1. Firstly, the records were only screened by one author. The impact of this was mitigated by taking a lenient approach to inclusion; articles were only excluded initially if the author was confident in their ineligibility. Where this was not the case, fellow authors were consulted to reach a consensus.
2. Secondly, a number of studies included in this review were found via citation and reference searching and were not captured as part of the initial search strategy. These studies were missed by the search strategy due to several factors; some did not state in the title or abstract that they included temporal data while some did not mention health records. Two terms were identified from these results that can be used to describe longitudinal data: ‘sequential’ and ‘trajectory’. While we are confident the most significant studies in the area were found, inclusion of these terms could have made the search strategy more comprehensive.
3. This review did not quantify the retrieved works as precisely as the framework described by Lauritsen [25] as this granularity would have impeded the ability to compare similar studies.
4. Finally, this review does not comment on the relative performances of each of the methods due to the heterogeneity of applications and datasets. The review can also not provide an answer as to whether longitudinal methods improve upon cross-sectional methods as this was rarely evaluated in the studies and is likely to be problem dependent.

## Conclusion

This review found a range of techniques that have been applied to longitudinal EHRs, including engineering of trend features and RNN based models. These models were used to predict a range of cancers, but most commonly pancreatic or colorectal cancer. Minimising the risk of bias is vital to ensuring progress towards deployable research. Key areas of potential bias were found, often relating to selection of cohorts and analysis of data. Potential mitigation strategies for these include sufficient follow-up of control populations and robust evaluation of model performance. Future research in this area should aim to evaluate prediction models with a range of temporal windows to find optimal timelines for application of the model; this analysis was rarely present in retrieved studies. To assist in the progression of these models from exploratory research to clinical practice, researchers should aim for clear reporting of methods, adhering to available taxonomies and reporting guidelines.

## Supplementary Information

Additional File 1. This file contains the search terms used for each of the databases.

Additional File 2. This file contains the overall data extraction table used for charting in the review.

Additional File 3. This file contains the domain-level PROBAST judgements for each study.

Additional File 4. This file contains extracted items relating to the reproducibility of the studies.

Additional File 5. This file contains the extracted data items describing the feature engineering and sequential input methods of the studies.

Additional File 6. Completed PRISMA-ScR checklist.

## Data Availability

The dataset supporting the conclusions of this article are included within the article and its additional files.
