# Supplementary material for: Artificial intelligence methods applied to longitudinal data from electronic health records for prediction of cancer: a scoping review
Source: BMC Med Res Methodol. 2025 Jan 28;25:24. doi: 10.1186/s12874-025-02473-w (PMC11773903; doi:10.1186/s12874-025-02473-w)

Additional File 1

# Search Terms

**IEEE**

("Machine learning" OR ML OR "Artificial Intelligence" OR AI OR "Deep Learning" OR "Data mining" OR "feature engineering" OR algorithm OR "neural network" OR "deep network" OR "convolutional network" OR "recurrent network" OR "graph network" OR transformer OR "support vector" OR "nearest neighbour" OR "gradient boost" OR XGBoost) AND (Predict* OR "Early detect*" OR diagnos* OR "risk stratif*" OR "clinical decision" OR Forecast*) AND ("Electronic health record*" OR "electronic patient record*" OR EHR OR EPR OR "Electronic Medical Record*" OR EMR) AND (Temporal OR Time-series OR longitudinal OR "Repeated Measures" OR "repeat measure" OR time-vary* OR time-dependent) AND (cancer OR neoplasm OR malignancy OR malignant OR carcinoma OR tumor OR tumour OR leukaemia OR lymphoma OR sarcoma OR myeloma)

N.B. IEEE has a limit on wildcards.

**SCOPUS**

TITLE-ABS-KEY ( "machine learning" OR ml OR "artificial intelligence" OR ai OR "deep learning" OR "data mining" OR "feature engineering" OR algorithm  OR "neural network" OR "deep network" OR "convolutional network" OR "recurrent network" OR "graph network" OR transformer OR "support vector" OR "nearest neighbour" OR "gradient boost" OR XGBoost) AND TITLE-ABS-KEY ( predict* OR "early detect*" OR diagnos* OR "risk stratif*" OR "clinical decision" OR forecast* ) AND TITLE-ABS-KEY ( "electronic health records" OR "electronic patient records" OR ehr OR epr OR emr OR "electronic medical records" ) TITLE-ABS-KEY ( temporal OR time-series OR longitudinal OR "repeat* measure*" OR "time-var*" OR "time-dependent" ) AND TITLE-ABS-KEY ( cancer OR neoplasm OR malignan* OR carcinoma OR lymphoma OR tumor OR tumour OR leukaemia OR myeloma OR sarcoma )

**EMBASE**

(("machine learning" or "artificial intelligence" or "AI" or "deep learning" or "data mining" or "feature engineering" or algorithm OR "neural network" OR "deep network" OR "convolutional network" OR "recurrent network" OR "graph network" OR transformer OR "support vector" OR "nearest neighbour" OR "gradient boost" OR XGBoost) and (predict* or "early detect*" or diagnos* or "risk stratif*" or "clinical decision" or forecast*) and ("electronic health record*" or "electronic patient record*" or "EHR" or "EPR" or "electronic medical record*" or "EMR") and (temporal or time-series or longitudinal or "repeat* measure*" or "time-vary*" or time-dependent) and (cancer or neoplasm or malignan* or carcinoma or tumour or tumor or leukaemia or lymphoma or sarcoma or myeloma))

**PUBMED**

("machine learning" OR "artificial intelligence" OR "AI" OR "deep learning" OR "data mining" OR "feature engineering" OR algorithm OR neural network* OR deep network* OR convolutional network* OR recurrent network* OR graph network* OR transformer OR support vector* OR nearest neighbour* OR gradient boost* OR XGBoost) AND (predict* OR "early detect*" OR diagnos* OR "risk stratif*" OR "clinical decision" OR forecast*) AND ("electronic health record*" OR "electronic patient record*" OR "EHR" OR "EPR" OR "electronic medical record*" OR "EMR") AND (temporal OR time-series OR longitudinal OR "repeat* measure*" OR "time-vary*" OR time-dependent) AND (cancer OR neoplasm or malignan* or carcinoma or tumour or tumor or leukaemia or lymphoma or sarcoma or myeloma)

**Web of Science**

( ALL= ( "machine learning" OR ml OR "artificial intelligence" OR ai OR "deep learning" OR "data mining" OR "feature engineering" OR algorithm OR neural network* OR deep network* OR convolutional network* OR recurrent network* OR graph network* OR transformer OR support vector* OR nearest neighbour* OR gradient boost* OR XGBoost) AND ALL= ( predict* OR "early detect*" OR diagnos* OR "risk stratif*" OR "clinical decision" OR forecast* ) AND ALL= ( "electronic health records" OR "electronic patient records" OR ehr OR epr OR emr OR "electronic medical records" ) AND ALL= ( temporal OR time-series OR longitudinal OR "repeat* measure*" OR "time-var*" OR "time-dependent" ) AND ALL= (cancer OR neoplasm or malignan* or carcinoma or tumour or tumor or leukaemia or lymphoma or sarcoma or myeloma))

**MEDLINE:**


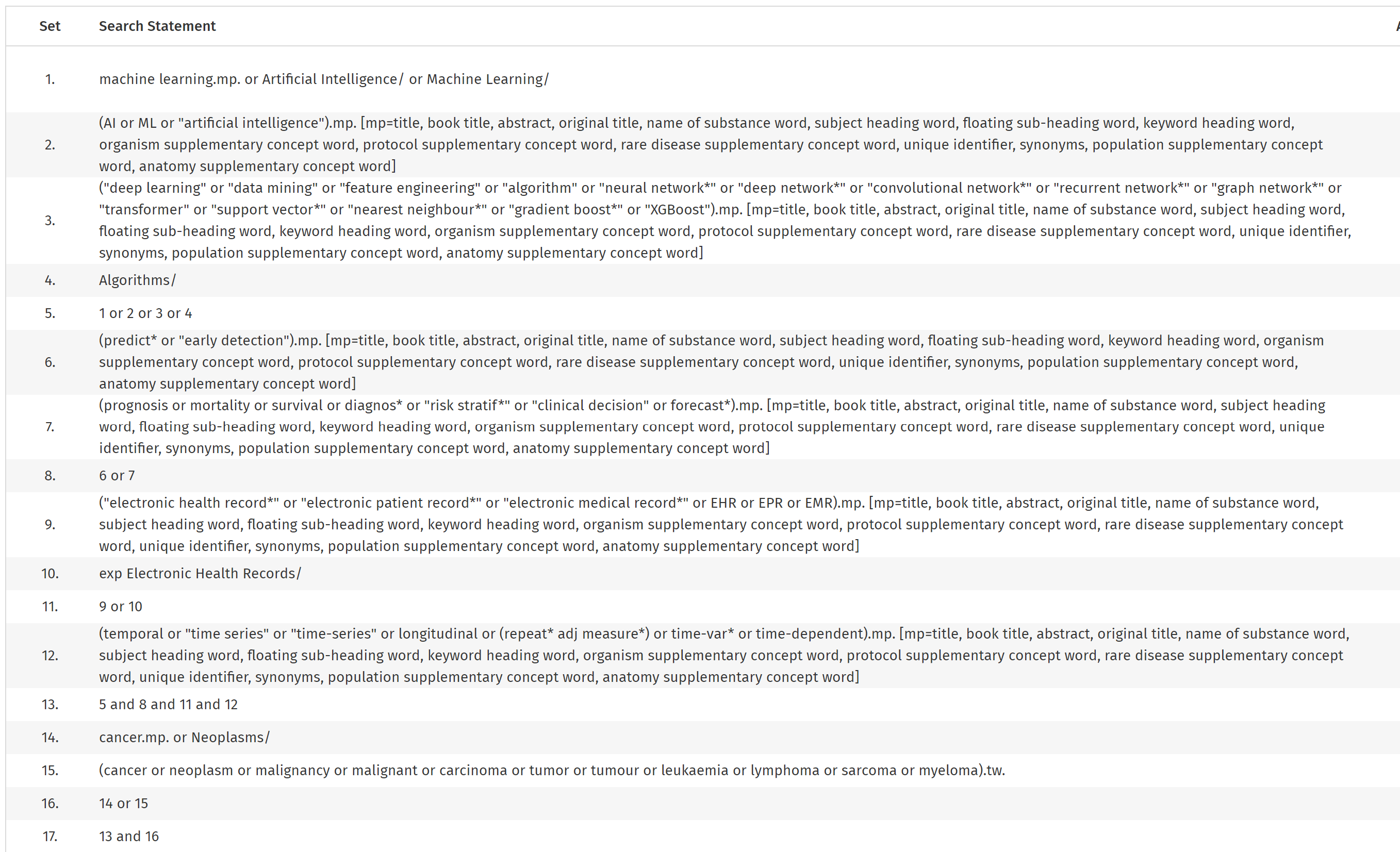

Supplement: Supplementary file 1 — Additional File 1. This file contains the search terms used for each of the databases. [file 12874_2025_2473_MOESM1_ESM.docx]
